# Supplementary material for: Cadmium Stabilization and Redox Transformation Mechanism in Maize Using Nanoscale Zerovalent-Iron-Enriched Biochar in Cadmium-Contaminated Soil
Source: Plants (Basel). 2022 Apr 14;11(8):1074. doi: 10.3390/plants11081074 (PMC9024939; doi:10.3390/plants11081074)
Supplement: Supplementary file 1 [file plants-11-01074-s001.zip › plants-1649447-supplementary.pdf]

**Supporting Information**

*Research article*

# Cadmium–stabilization and redox transformation mechanism in maize using nanoscale zerovalent−iron enriched biochar in Cd contaminated soil

**Sehar Razzaq[1,2], Beibei Zhou[1*], Adnan Raza Altaf[4], Saddam Hussain[3], Ghous Bakhsh[5], Zia Ur Rehman[2], Amir Maqsood[2], Zhenshi Zhang[6], Qiang Yang[6]**

1. State Key Laboratory of Eco-hydraulics in Northwest Arid Region of China, Xi'an University of Technology, Xi'an 710048, China
2. Institute of Soil & Environmental Sciences, University of Agriculture, Faisalabad, 38040, Pakistan.
3. Department of Agronomy, University of Agriculture, Faisalabad, 38040, Pakistan.
4. School of Chemical Engineering, Northwest University, Xi'an, 710069, China.
5. Training and Publicity, Agriculture Extension, Dera Allah Yar Jaffarabad Balochistan, 08289, Pakistan.
6. Power China Northwest Engineering Corporation Limited, Xi'an, Shaanxi,China

* Correspondence: happyangle222@aliyun.com (B. Zhou)

**Sorbent Characterization:** The sorbents' specific surface area and pores characteristics were determined by $N_2$ adsorption/desorption isotherm system at -196 °C (Quantachrome Nova 2000e, USA). The crystal structures on the sorbents were analyzed by X-ray diffractor-meter (XRD Philips APD 3720), and the position was 2°. The surface morphology of sorbent was analyzed by scanning electron microscope (SEM, JOEL 7100F). The sorbents surface functional groups were characterized before and after modification by Fourier transform infrared spectrometer (FT−IR, T9000 Perkin Elmer, USA), and the scanned IR spectral range was 450 to 4000 $cm^{-1}$.
